# Electrochemical Characterization of Electrodeposited Copper in Amine CO_2_ Capture Media

**DOI:** 10.3390/ma17081825

**Published:** 2024-04-16

**Authors:** Corentin Penot, Kranthi Kumar Maniam, Shiladitya Paul

**Affiliations:** 1 Materials Innovation Centre, School of Engineering, University of Leicester, Leicester LE1 7RH, UK; cp473@leicester.ac.uk (C.P.); km508@leicester.ac.uk (K.K.M.); 2 Materials Performance and Integrity Technology Group, TWI, Cambridge CB21 6AL, UK

**Keywords:** electrodeposition, copper, electrochemical CO_2_ reduction, corrosion, amine capture, carbon capture

## Abstract

This study explores the stability of electrodeposited copper catalysts utilized in electrochemical CO_2_ reduction (ECR) across various amine media. The focus is on understanding the influence of different amine types, corrosion ramifications, and the efficacy of pulse ECR methodologies. Employing a suite of electrochemical techniques including potentiodynamic polarization, linear resistance polarization, cyclic voltammetry, and chronopotentiometry, the investigation reveals useful insights. The findings show that among the tested amines, CO_2_-rich monoethanolamine (MEA) exhibits the highest corrosion rate. However, in most cases, the rates remain within tolerable limits for ECR operations. Primary amines, notably monoethanolamine (MEA), show enhanced compatibility with ECR processes, attributable to their resistance against carbonate salt precipitation and sustained stability over extended durations. Conversely, tertiary amines such as methyldiethanolamine (MDEA) present challenges due to the formation of carbonate salts during ECR, impeding their effective utilization. This study highlights the effectiveness of pulse ECR strategies in stabilizing ECR. A noticeable shift in cathodic potential and reduced deposit formation on the catalyst surface through periodic oxidation underscores the efficacy of such strategies. These findings offer insights for optimizing ECR in amine media, thereby providing promising pathways for advancements in CO_2_ emission reduction technologies.

## 1. Introduction

Mitigating climate change necessitates urgent action to reduce CO_2_ emissions. Achieving net zero CO_2_ emissions by 2050 is critical to limit the temperature rise to below 1.5 °C, in line with the Paris Agreement (COP21) objectives [1]. To address this challenge, the development of CO_2_ capture technologies is a priority. Currently, the most advanced industrial technology employs aqueous amine solutions such as monoethanolamine (MEA), methyldiethanolamine (MDEA), and 2-amino-2-methylpropanol (AMP) for CO_2_ capture. This process entails direct CO_2_ capture from the flue gas stream and its subsequent release by heating the CO_2_-rich amine capture media in a desorber unit at approximately 120 °C [2]. The regenerated amine then re-enters the capture cycle. However, the regeneration step, accounting for up to 30% of the total plant energy output, is highly energy-intensive [3]. This issue may be overcome by regenerating the CO_2_-rich amine media using electrochemical CO_2_ reduction (ECR) instead. This approach features the direct transformation of amine-CO_2_ adducts, such as carbamates, into valuable chemicals [4]. The seamless integration of amine-based CO_2_ capture and ECR demonstrates good synergy. This approach not only bypasses challenges related to CO_2_ transportation and storage but also eradicates the necessity for regenerating capture media through the thermal release of molecular CO_2_ [5].

ECR is an emerging field, evolving independently from carbon capture technologies. It presents significant synergies with other climate change challenges, notably in storing surplus renewable electricity. ECR converts excess electricity into valuable energy-dense products like CO, ethylene, and ethanol. The output product spectrum depends on the applied potential, catalyst, and cell configuration [6]. Copper is unique in promoting valuable C_2_+ products like ethylene and is, therefore, widely employed as a catalyst [7]. Achieving economic viability in ECR hinges on meeting specific performance benchmarks, including current density, Faradaic efficiency (FE), energy efficiency (EE), and stability [8]. Recent research has made significant strides in improving current density, FE, and EE. Notably, current densities over 1 A cm^−2^ with 45% EE for ethylene production using copper gas diffusion electrodes (GDEs) have been achieved [9]. Ongoing efforts aim to enhance selectivity toward C_2_+ products through advanced copper-based catalysts [10,11,12]. However, stability improvements remain a less explored yet critical aspect for viable ECR. Copper catalysts often undergo rapid degradation due to surface reconstruction, poisoning, and carbonate salt precipitation [13]. Studies by Huang et al. [14] and Simon et al. [15] show that cathodic potential drives copper catalyst surface reconstruction and refaceting. This process results in decreased selectivity towards C_2_+ products, undermining long-term catalyst performance [16,17,18,19]. Another challenge for stable ECR is carbonate salt precipitation on the catalyst surface [20,21,22]. The local high pH near the electrode, due to HO^−^ production in the CO_2_ reduction reaction, facilitates carbonate salt formation through reaction with cations.

Recent advancements in electrochemical CO_2_ reduction (ECR) have highlighted pulse ECR as a method to enhance long-term process performance [23]. Pulse ECR involves interspersing short anodic segments with longer cathodic ones to regenerate the catalytic properties of copper by inducing oxidation. Obasanjo et al. [24] demonstrated that this oxidation process reactivates C_2_+ active sites by removing Cu-OH, which gradually deactivates the catalyst. Xu et al. [21] successfully employed pulse ECR to sustain high Faradaic efficiency (FE) towards C2+ products over 36 h, observing that Cu(I) oxide formation is advantageous for C2+ product formation and can be regenerated through anodic pulsing. Similarly, Zhang et al. [25] found that a combination of Cu(0) and Cu(I) states favors C_2_+ products, maintaining a high FE (70%) towards ethylene across 145 h of operation using pulse ECR. Complementary studies have shown that appropriate pulse strategies can enhance and modulate the long-term selectivity of ECR [26,27], while also mitigating catalyst poisoning [28] and carbamate salt formation [21,29].

Research on ECR, including pulse ECR has largely focused on using aqueous carbonate (KOH + CO_2_) or bicarbonate (KHCO_3_ + CO_2_) solutions. Increasing efforts are now directed towards integrating ECR with amine-based CO_2_ capture. Various studies have reported carbamate reduction to CO [30], formate [31,32], or both [33] using metal electrodes like copper [30,32]. However, the long-term stability of these catalytic properties remains unexplored. Additionally, the impact of amines on copper corrosion is a growing concern [5], with some studies suggesting significant copper degradation in MEA under halted cathodic potential [34], which necessitates further investigation.

This study is part of the larger CoCaCO2la project, which aims to demonstrate an isothermal capture and conversion intensified process, where CO2 release, conversion, and capture media regeneration are performed in the electrolyzer, enabling an isothermal loop for carbon capture. To promote scalability, affordable and low lead time processes, such as electrodeposition, were investigated for Cu catalyst production.

In this study, we explore the electrochemical and corrosion properties of electrodeposited copper in amine-based electrolytes under CO_2_-lean and -rich conditions. We also examine the stability of the copper catalyst under cathodic polarization relevant to CO_2_ reduction. Different pulse polarization strategies are tested to assess the impact of pulse ECR in amine media.

## 2. Materials and Methods

### 2.1. Specimen Preparation and Characterisation

Copper was electrodeposited onto a copper substrate using a method detailed in the Supplementary Materials. Following electrodeposition, specimens were selectively masked with resin provided by Belzona Ltd. (Hawarden, UK, reference 1395) resin, resulting in an exposed surface area of approximately 1 cm^2^ for each specimen. To characterize the surfaces of these specimens, an optical microscope BX41M-LED from Olympus (Hachioji, Japan) and a Sigma 1455EP scanning electron microscope (SEM) from Ziess (Oberkochen, Germany) were employed.

### 2.2. Amine Media

Four amine-based media, commonly employed for CO_2_ capture, were evaluated:

- 30 wt.% Monoethanolamine (MEA), CAS: 141-43-5, a primary amine extensively utilized for CO_2_ capture [2].
- 37 wt.% Methyldiethanolamine (MDEA) CAS: 105-59-9, a tertiary amine.
- 30 wt.% MDEA + 21 wt.% piperazine (PZ), CAS: 110-85-0, generally used to improve capture kinetics [35].
- 30 wt.% 2-Amino-2-methyl-1-propanol (AMP), CAS: 124-68-5, a sterically hindered primary amine known for its elevated CO_2_ absorption capacity [36].

The anolyte for these experiments was 0.5 M potassium chloride (KCl) solution. Additionally, 0.5 M KCl was incorporated into the amine solutions to enhance their electrical conductivity. All chemicals used in solution preparation were of laboratory grade and supplied by Merck Life Science UK Ltd. (Gillingham, UK).

In subsequent discussions and analyses, these solutions are referred to as MEA, MDEA, MDEA/PZ, and AMP for simplicity.

### 2.3. Electrochemical Tests

**Figure 1:**
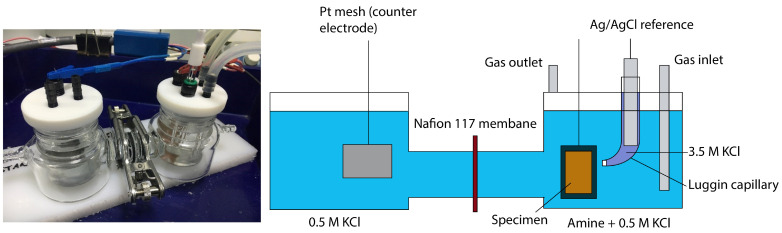
H-cell three-electrode setup for electrochemical testing.

Electrochemical experiments were conducted using a Biologic (Grenoble, France) VMP-300 potentiostat and a 100 mL H-cell sourced from Dek research (Hong Kong), as represented in Figure 1. The experimental setup featured anodic and cathodic compartments, separated by a Nafion 117 proton exchange membrane (Dupont, Wilmington, DE, USA) which underwent appropriate activation before use. The reference electrode comprised an Ag/AgCl in 3.5 M KCl, positioned in a Luggin capillary filled with 3.5 M KCl to isolate it from the amine electrolyte. The capillary’s tip was situated 5 mm from the specimen. A platinum mesh served as the counter electrode. The cell design included gas inlet/outlet provisions for purging the solution with CO_2_. Each specimen was kept at −0.5 V relative to the Open Circuit Potential (OCP) for 5 min before testing to remove the air-formed oxide layer.

Linear polarization resistance (LPR), potentiodynamic polarization (PDP), and cyclic voltammetry (CV) tests were performed after recording and stabilizing the OCP for 1 h. To ensure reproducibility, each measurement was conducted in triplicate. LPR results are presented in Supplementary Materials Figure S6. The test solutions were maintained at ambient laboratory conditions (20 ± 2 °C) and could be CO_2_-purged as required. LPR tests utilized a potential offset of ±20 mV relative to OCP at a scan rate of 0.125 mV s^−1^. PDP involved potential sweeping from −0.2 V to +0.2 V versus OCP at a rate of 10 mV min^−1^. CV scans were conducted at 20 mV s^−1^ scan rate, reversing the scan when the current density reached |10| mA cm^−2^ for both cathodic and anodic polarization.

**Figure 2:**
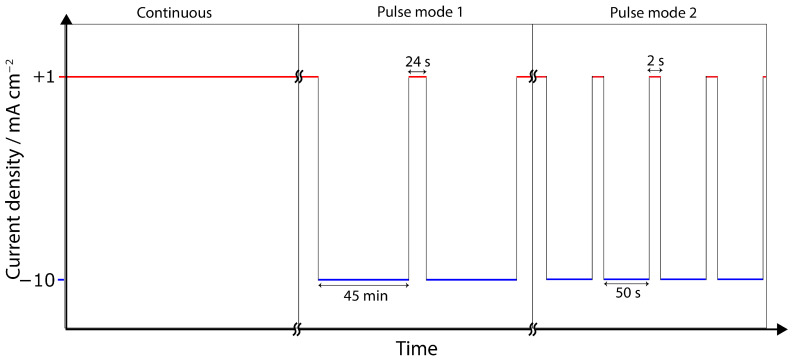
Three modes of chronopotentiometry: (1) continuous polarization at −10 mA cm^−2^ for 60 h; (2) pulse mode 1, 45 min at −10 mA cm^−2^ for 24 s at +1 mA cm^−2^ repeated 80 times; (3) pulse mode 2, 50 s at −10 mA cm^−2^ for 2 s at +1 mA cm^−2^ repeated 4320 times.

Chronopotentiometry experiments were designed to simulate 60 h of ECR. Three strategies, which included alternating anodic and cathodic segments, were implemented as detailed in Figure 2. Anodic segments were conducted at +1 mA cm^−2^, while cathodic segments operated at −10 mA cm^−2^, cumulatively totaling 60 h for cathodic segments. Pulse mode 1 consisted of a 24 s anodic segment every 45 min, and pulse mode 2 involved a 2 s anodic segment every 50 s.

Electrochemical tests underwent iR compensation utilizing the potentiostat’s built-in feature, which is based on high-frequency impedance measurements. The average cell resistances determined for each media are systematically tabulated in Supplementary Materials Table S1.

## 3. Results

### 3.1. Electrochemical Characterisation of Electrodeposited Copper in Amine Media

**Figure 3:**
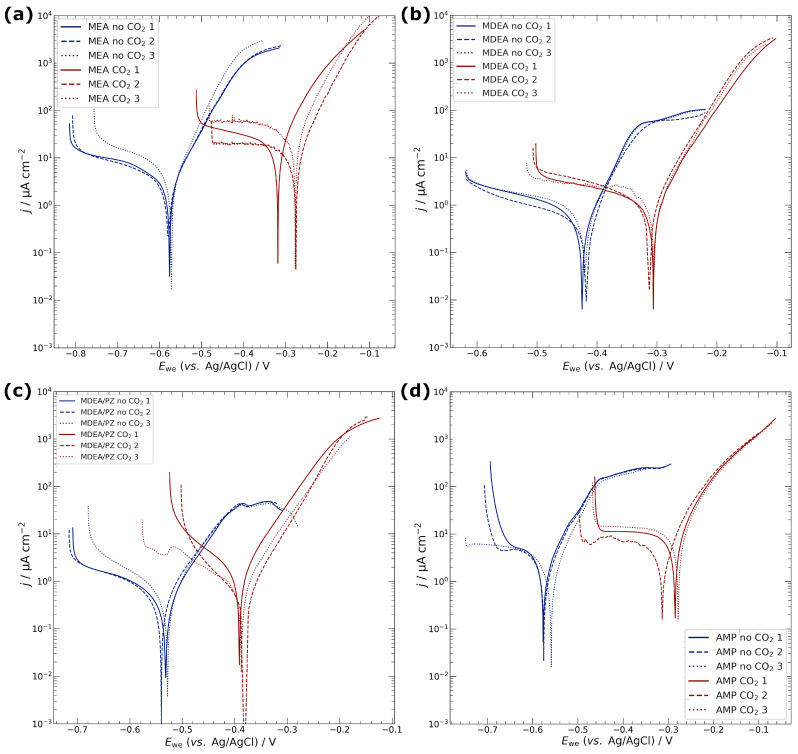
Potentiodynamic polarization of copper specimens in the different amine-based capture media. (**a**) MEA, (**b**) MDEA, (**c**) MDEA/PZ, and (**d**) AMP. Scan rate 10 mV min^−1^.

Potentiodynamic polarizations were performed in triplicate under both CO_2_-free and CO_2_-purged conditions across various amine media and are shown in Figure 3. The Tafel extrapolation method was employed to estimate the corrosion current density (*j*_corr_) [37]; the full set of Tafel extrapolation is provided in Supplementary Materials, Figures S2–S5. The average *j*_corr_ values, together with the corresponding corrosion potentials (*E*_corr_), are compiled in Table 1.

In environments devoid of CO_2_, *j*_corr_ values for all amines were lower compared to 0.5 M KCl. Specifically, MDEA and MDEA/PZ exhibited reduced *j*_corr_ values relative to MEA and AMP, with the latter two showing *j*_corr_ values comparable to KCl. Upon introducing CO_2_, a notable shift in *E*_corr_ towards more anodic potentials occurred for all amines, attributable to the dissolution of CO_2_ and the formation of carbonate/bicarbonate species. Concurrently, the introduction of CO_2_ resulted in an increase in *j*_corr_ across all amines. The relative order of *j*_corr_ values remained consistent with the CO_2_-free cases: MEA and AMP exhibited higher *j*_corr_, exceeding that of KCl, while MDEA and MDEA/PZ maintained significantly lower values. Polarization resistance (*R*_p_) data, derived from LPR measurements, were consistent with *j*_corr_. Lower *R*_p_ values, indicative of higher corrosion rates, were observed for CO_2_-loaded MEA and AMP, whereas CO_2_-free MDEA and MDEA/PZ demonstrated higher resistances.

**Figure 4:**
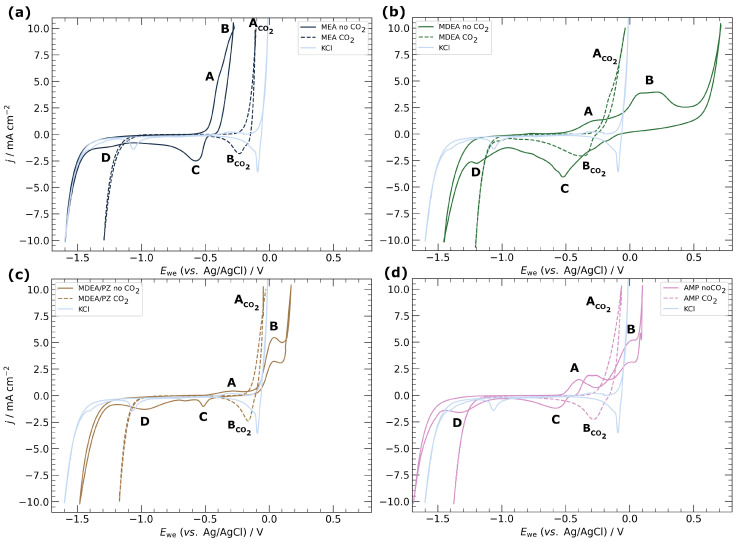
Cyclic voltammetry of electrodeposited copper specimens in different amine-based capture media under CO_2_-lean and CO_2_-rich conditions: (**a**) MEA, (**b**) MDEA, (**c**) MDEA/PZ, and (**d**) AMP. Scan rate 20 mV s^−1^.

CV scans, presented in Figure 4, reveal distinct electrochemical behaviors of copper in various amine media. For comparative purposes, CV experiments were also conducted in 0.5 M KCl. In CO_2_-lean amine media, two oxidation peaks (A and B) and two reduction peaks (C and D) were identified. Peak onset potentials are tabulated in Table 2. It is well established that copper oxidizes to form either Cu(I) or Cu(II) [38]. During the anodic sweep, the initial peak (A) is attributed to the formation of Cu(I), while the subsequent peak (B) corresponds to Cu(II) oxidation, both manifesting as copper oxides/hydroxides and their hydrated forms. The oxidation current increase beyond peak B signifies the formation of soluble copper ions such as CuO_2_^−^ until the onset of oxygen evolution at more anodic potentials. The onset potential for peak A remains relatively consistent across all CO_2_-free amine media, approximately at 0.45 V. However, the onset for peak B varies significantly, indicating a more complex oxidation mechanism, which is in line with previous findings in KOH solutions [39]. In MEA, the lowest onset potential leads to the overlapping of peaks A and B. In contrast, MDEA, MDEA/PZ, and AMP exhibit distinct B peaks at more anodic potentials, at −0.27 V, −0.15 V, and −0.27 V, respectively. Peaks C and D are associated with the reduction in Cu(I) and Cu(II) species, while the highest cathodic current surge is attributed to the hydrogen evolution reaction (HER).

In CO_2_-rich amine media, only two peaks, labeled A_CO2_ and B_CO2_, are evident. Peak A_CO2_ could represent the overlap of peaks A and B, indicating the concurrent formation of both Cu(I) and Cu(II) species, or it might signify the exclusive formation of Cu(II) species. The former scenario seems more probable, as the overlapping of peaks A and B has been previously reported in high pH conditions [39], likely influenced by CO_2_ purging. The onset oxidation potentials in various amines are closer in the CO_2_-rich environment, recorded at −0.21 V, −0.27 V, −0.17 V, and −0.25 V for MEA, MDEA, MDEA-PZ, and AMP, respectively. This observation underscores the significant impact of the presence of CO_2_ on copper oxidation and supports previous PDP and LPR data. Copper oxidizes more readily in CO_2_-rich media, as evidenced by a steeper increase in oxidation current at more cathodic potentials, except for MEA, which exhibits a slightly higher oxidation onset potential in CO_2_-rich conditions. In CO_2_-rich amines, the most anodic current is either due to HER or the CO_2_ reduction reaction (CO_2_RR) and is shifted towards more anodic potentials. This shift suggests that CO_2_RR may have a more anodic onset potential than HER, leading to an observable increase in current density on the CV scan for CO_2_-purged media.

### 3.2. Chronopotentiometry

**Figure 5:**
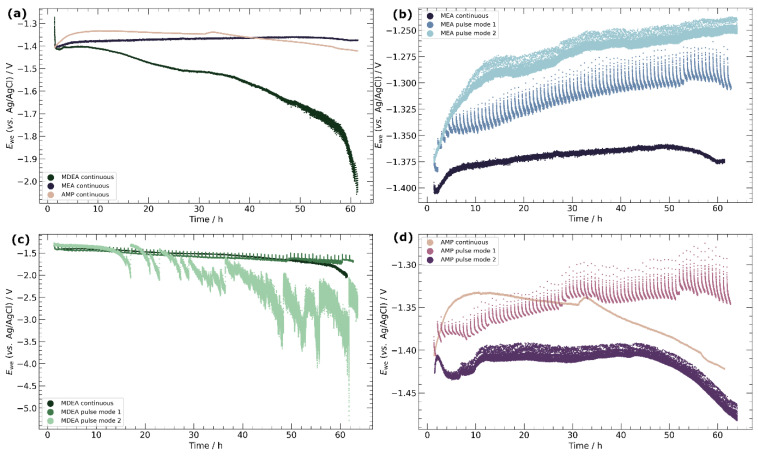
Cathodic chronopotentiometry of electrodeposited copper specimen in 30 wt.% MEA, 5 m MDEA, and 30 wt.% AMP solutions purged with CO_2_. (**a**) Continuous cathodic polarization; (**b**–**d**) continuous and pulse mode 1 and 2 in MEA, MDEA, and AMP, respectively. Target current density was −10 mA cm^−2^.

**Figure 6:**
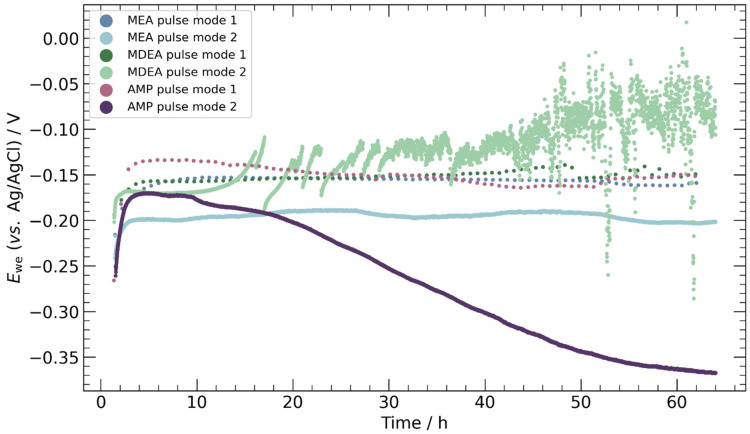
Anodic segments from chronopotentiometry of electrodeposited copper specimen in 30 wt.% MEA, 5 m MDEA, and 30 wt.% AMP solutions purged with CO_2_. Target current density was +1 mA cm^−2^. Only the last data point of each cycle is represented for clarity.

Specimens underwent cathodic polarization for a cumulative duration of 60 h, employing varied pulse strategies. These strategies encompassed continuous cathodic polarization, pulse mode 1, and pulse mode 2, as detailed in Figure 2. The potential as a function of time was closely monitored, with the corresponding cathodic chronopotentiometry results displayed in Figure 5. Anodic chronopotentiometry results are similarly detailed in Figure 6.

Chronopotentiometry results for continuous cathodic polarization over 60 h in MEA, MDEA, and AMP are illustrated in Figure 5a. In MDEA/PZ, this experiment was hindered by the formation of carbonate salts, which obstructed the CO_2_ gas inlet after only 2–3 h. Similar carbonate salt precipitation was observed in MDEA, leading to an unstable cathodic potential that progressively shifted towards more cathodic values, as depicted in Figure 5a. In contrast, the potentials in AMP demonstrated greater stability, albeit with a slight decrease over time. Notably, MEA exhibited the most stable potential, concluding the experiment at a potential 20 mV higher than the initial value.

The influence of pulse strategies on potential evolution for the various amine solutions is highlighted in Figure 5b–d. Employing a lower frequency pulse strategy (pulse mode 1) consistently improved potential stability across all amine solutions. In the case of MDEA, this strategy resulted in a stable potential decrease beyond 55 h, contrasting with the trend observed under continuous polarization. For MEA and AMP, pulse mode 1 induced a gradual electropositive shift in potential, reaching increments of +70 mV and +65 mV from their initial values, respectively. The adoption of a higher frequency of anodic segments (pulse mode 2) demonstrated diverse outcomes. In MEA, pulse mode 2 more significantly improved potential stability compared to pulse mode 1, achieving an increase of +125 mV compared to the initial value. In contrast, for AMP, the potential under pulse mode 2 initially remained stable but began to decline after 45 h, aligning more closely with the behavior observed under continuous polarization. For MDEA, the potential during pulse mode 2 rapidly deteriorated and became extremely unstable upon the onset of carbonate salt precipitation.

Anodic chronopotentiometry results for the various pulse strategies are shown in Figure 6. Three distinct behaviors emerged: stable, erratic, and decreasing. A stable potential was noted in all specimens subjected to pulse mode 1, as well as in MEA under pulse mode 2. The potential required to reach a current density of +1 mA cm^−2^ hovered around −0.15 V for pulse mode 1 and −0.2 V for pulse mode 2 in MEA. This variance is attributed to surface charging/discharging phenomena, necessitating more anodic potentials to offset the declining discharging current with an elevated Faradaic oxidation current for longer anodic durations. Since pulse mode 2 features shorter durations, the proportion of Faradaic current is reduced, hence less anodic potentials are sufficient to sustain the target current density.

In the case of MDEA and AMP under pulse mode 2, the anodic potential exhibited instability, albeit with markedly different behaviors. In MDEA using pulse mode 2, an erratic potential was observed beyond 15 h, likely caused by the intermittent formation and detachment of carbonate salts on the catalyst surface. In contrast, AMP during pulse mode 2 exhibited a continuous cathodic shift in anodic potential over time.

**Figure 7:**
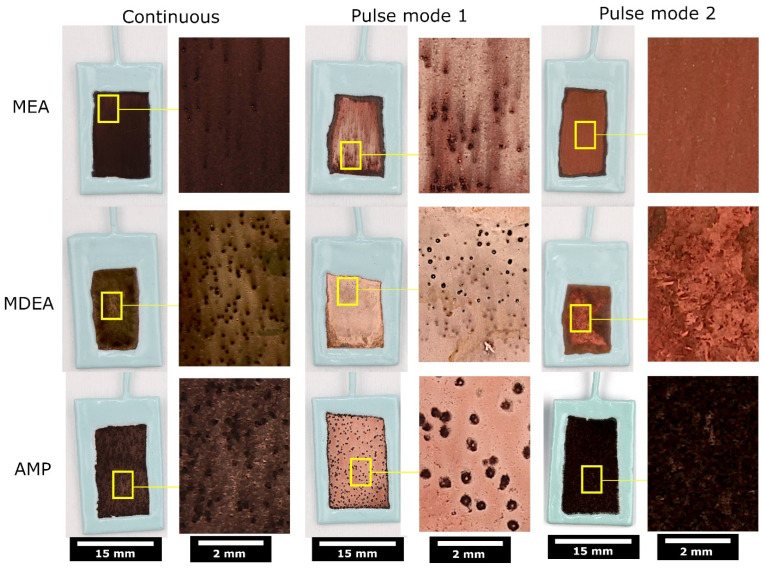
Optical macrographs of catalysts after chronopotentiometry.

The post-chronopotentiometry visual appearance of copper catalysts exhibits marked variations contingent on the polarization mode, as illustrated in Figure 6. Continuously polarized specimens display darker surfaces, whereas those subjected to pulse modes generally manifest ‘cleaner’ copper surfaces, with the exception of AMP under pulse mode 2. When correlating the visual observations of the catalysts after chronopotentiometry with the anodic potential data from Figure 6, it becomes evident that periodic oxidation facilitated by pulse modes effectively mitigates the formation of dark deposits. The pronounced dark deposit observed in AMP under pulse mode 2 indicates inadequate oxidation of the copper catalyst. Indeed, several factors can contribute to a cathodic shift in potential when maintaining a target anodic current density, such as a reduction in active surface area, a shift in oxidation onset potential, or an increased charging of the interface reducing the proportion of Faradaic currents required to achieve the target. The observed potential diminution for AMP pulse mode 2 is presumably attributed to the latter, as post-test surface characterization revealed a dark deposit akin to that observed following continuous polarization, as shown in Figure 7. This suggests that the intended oxidation of copper was not effectively achieved, pointing to surface charging as the underlying cause for the diminished oxidation. Under these specific test conditions, an anodic duration of 2 s proved insufficient for achieving copper oxidation in CO_2_-rich AMP. These findings underscore the importance of cyclic copper oxidation in preventing the formation of such dark deposits.

Artefacts resulting from a gas generation at the catalyst surface, characterized by black spots with occasional dark trails directed upward, are discernible in specimens under continuous and pulse mode 1 polarization. However, these artefacts are absent in samples subjected to pulse mode 2, where no such phenomena are observed.

**Figure 8:**
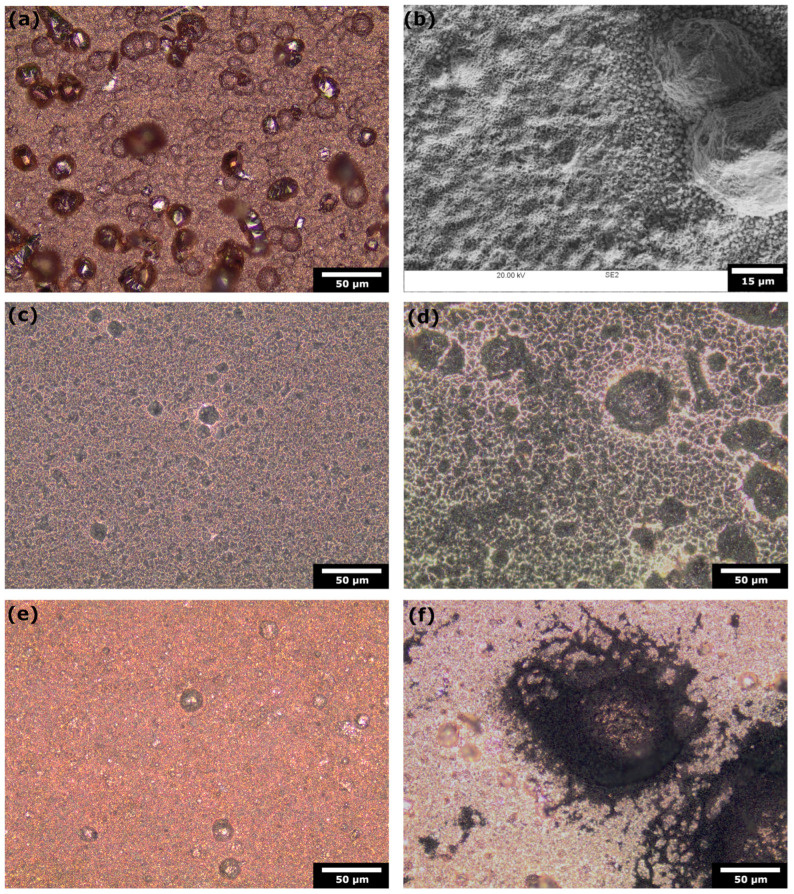
Optical micrographs and SEM of electrodeposited copper catalyst (**a**,**b**) prior to chronopotentiometry; (**c**,**d**) after continuous chronopotentiometry in MEA and AMP, respectively; (**e**) after pulse mode 2 in MEA; (**f**) after pulse mode 1 in AMP.

Higher magnification images of the catalyst surface are provided in Figure 8. Prior to chronopotentiometry, as shown in Figure 8a,b, the surface primarily consists of pure copper, characterized by an uneven topology with sporadic overgrown protrusions, ranging from 15 µm to 35 µm in diameter. After continuous polarization, the formation of a dark deposit is apparent, leaving only a thin network of exposed copper (Figure 8c,d). Conversely, in MEA under pulse mode 2 (Figure 8e), the surface displays no discernible dark deposits, and the integrity of the surface is notably preserved. An instance of the gas generation artefact, observable in MEA under pulse mode 1 (Figure 8f), exhibits a dark deposit in the shape of a ring, approximately 100 µm in diameter, with a center of exposed copper corresponding to the cathodic site.

## 4. Discussion

### 4.1. Corrosion of Copper Catalyst in Amine Media

Corrosion rates were calculated from the corrosion current density (*j*_corr_) in accordance with ASTM G102 guidelines [40]. The derived corrosion rates, along with the time necessary to dissolve a 50 µm copper deposit (the estimated thickness based on weight measurements), are tabulated in Table 3 These calculations reveal that the complete dissolution of the copper deposit, under the presumption of exclusive Cu(I) formation and in the absence of any passivation, would necessitate 56 days in the most severe scenario (CO_2_-enriched MEA). In the case of Cu(II) formation, the time extends to 112 days. It is critical to recognize that the hypothesis of no passivation is valid only immediately after the termination of the cathodic current, as an oxide protective layer is expected to form subsequently. Thus, these calculations are pertinent mainly for approximating the cumulative switch-off duration tolerable by the catalyst. Accordingly, a cumulative switch-off time threshold of 56 days is considered more than adequate. Therefore, the free corrosion of copper in the amine media does not constitute a limiting factor for the catalyst’s operational lifespan.

The influence of corrosion-induced surface alterations on the catalyst’s efficiency, including aspects such as selectivity, FE, and EE, still requires further investigation. However, existing literature, including findings by [41], indicates that copper oxides favor the reduction in CO_2_ into C_2_+ products, suggesting that corrosion may not pose immediate concerns for catalyst functionality.

### 4.2. Choice of Amine Capture Media for ECR

CO_2_ capture in amine media is primarily governed by two processes. The initial process involves the formation of carbamate, as described by reaction (1). Subsequently, the formed carbamate undergoes hydrolysis into bicarbonate, delineated in reaction (2). The prevalence of these reactions is contingent on the amine type. Non-sterically hindered primary amines, such as MEA, tend to form stable carbamate; thus, reaction (1) predominates, leading to almost complete CO_2_ capture in the form of carbamate [42]. While this mechanism offers rapid capture kinetics, it necessitates higher energy for CO_2_ release.

In sterically hindered amines like AMP, the carbamate bond is less stable [36,43], enhancing the carbamate hydrolysis reaction (reaction 2). This results in CO_2_ being stored as both carbamate and bicarbonate, combining the fast capture kinetics of reaction (1) with an increased CO_2_ absorption capacity due to the 1:1 stoichiometry between CO_2_ and the amine molecule in reaction (2), in contrast to the 2:1 ratio in reaction (1).

(1) $${CO}_{2}+2R_{1}R_{2}NH⇌R_{1}R_{2}N^{+}H_{2}$$

(2) $$R_{1}R_{2}N^{+}H_{2}+H_{2}O⇌R_{1}R_{2}NH+HCO_{3}^{-}$$

Tertiary amines, such as MDEA, do not form carbamate with CO_2_. Instead, they function as catalysts for CO_2_ hydration [44], leading to exclusive bicarbonate capture as per reaction (3). This mechanism offers a higher absorption capacity, albeit at a slower capture rate.

(3) $${CO_{2}+H_{2}O+R}_{1}R_{2}R_{3}N⇌HCO_{3}^{-}+R_{1}R_{2}R_{3}N^{+}H$$

Historically, capturing CO_2_ as bicarbonate has been favorable due to its greater absorption capacity and reduced thermal energy requirement for CO_2_ release. The findings presented here indicate that tertiary amine can lead to significant carbonate salt precipitation during ECR, as observed in MDEA and MDEA/PZ. In contrast, primary amines do not facilitate carbonate salt formation, as evidenced in MEA and AMP. Based on these observations, primary amines are emerging as more promising candidates for direct ECR applications. This is confirmed by chronopotentiometry results which showed greater stability for MEA.

### 4.3. Pulse ECR in Amine Media

Pulse chronopotentiometry findings clearly demonstrate that incorporating short anodic segments counteracts the cathodic shift in potential over time, often resulting in an anodic shift in cathodic potentials. This trend suggests an enhancement in the efficiency of reduction reactions, as evidenced by lower overpotentials needed to maintain the target current density. The formation of copper oxides during anodic segments is likely responsible for this anodic potential shift.

Beyond the advantageous potential shift, pulse ECR exhibits a pronounced ability to preserve the catalyst surface, effectively minimizing the formation of dark deposits. These outcomes highlight the necessity for optimization in pulse ECR to enhance results, such as maximizing potential shifts and reducing deposit formation, as exemplified by pulse mode 2 in MEA. The optimization process varies with the media used, as evidenced by the differences in outcomes for MEA and AMP under pulse mode 2. Key considerations for effective pulse ECR include ensuring adequate oxidation of the copper surface by setting a sufficiently long anodic step duration, counter to the approach seen in AMP under pulse mode 2. Additionally, the cathodic step duration should be short enough to prevent excessive deposit formation at gas generation sites, such as observed in AMP under pulse mode 1, which could be problematic to eliminate during subsequent anodic steps.

Carbonate salt formation arises from localized HO^−^ ion enrichment near the catalyst surface, a byproduct of the CO_2_ reduction reaction (CO_2_RR) [20]. Previous studies have suggested that pulse ECR can mitigate carbonate salt formation by curbing HO^−^ accumulation [21,29]. However, the pulse strategies evaluated were ineffective in preventing carbonate salt precipitation in MDEA-based capture media. This outcome implies that tertiary amines favor carbonate salt formation.

## 5. Conclusions

The investigation into the stability of electrodeposited copper catalysts for Electrochemical Reduction (ECR) in various amine media offers valuable insights into critical aspects of this process. The study underscores several key findings that advance our understanding and pave the way for future research endeavors:

- Corrosion is not a significant impediment to the catalyst’s longevity in amine media. Both computational modeling and experimental data corroborate that the inherent corrosion of copper in these conditions does not critically limit the operational lifespan of the catalyst. This insight alleviates concerns regarding the durability of copper catalysts in practical ECR applications.
- Primary amines, particularly MEA, demonstrate higher compatibility with ECR processes, characterized by the absence of carbonate salt precipitation and more stable potentials over time. This observation emphasizes the importance of considering the amine type in optimizing ECR performance and underscores the potential for tailored catalyst–amine combinations to improve efficiency.
- Pulse ECR demonstrated significant potential in improving ECR stability, manifested by a shift in cathodic potential and effective mitigation of deposits on the catalyst surface through periodic oxidation. This highlights the importance of exploring innovative operational strategies to augment the stability and efficiency of ECR processes.

Future investigations should prioritize delineating the specific products formed from the reduction in amine–carbamate adducts using electrodeposited copper catalysts. Understanding these reaction pathways is paramount for optimizing the ECR processes and expanding their industrial applications. Additionally, the effect of pulse strategies on ECR productivity needs exploration, particularly in understanding how the intermittent nature of these strategies influences the rate and efficiency of CO_2_ conversion.

## Figures and Tables

**Table 1:** Average corrosion potential (*E*_corr_), corrosion current density (*j*_corr_), and polarization resistance (*R*_p_) for electrodeposited copper specimens in different amine media.

Media	CO_2_	*E*_corr_/V	*j*_corr_/µA cm^−2^	*R*_p_/kΩ cm^2^
MEA	no	−0.58 ± 0.01	2.4 ± 0.1	3.8 ± 0.7
	yes	−0.29 ± 0.02	14.1 ± 6.9	0.7 ± 0.2
MDEA	no	−0.42 ± 0.01	0.3 ± 0.1	19.0 ± 4.4
	yes	−0.33 ± 0.03	1.0 ± 0.1	10.4 ± 0.5
MDEA/PZ	no	−0.53 ± 0.01	0.4 ± 0.1	18.3 ± 6.3
	yes	−0.39 ± 0.01	0.9 ± 0.6	12.6 ± 11.3
AMP	no	−0.57 ± 0.01	1.5 ± 0.3	5.6 ± 1.1
	yes	−0.29 ± 0.02	7.7 ± 1.3	1.0 ± 0.6
KCl	no	−0.20 ± 0.03	2.4 ± 0.6	3.7 ± 1.1

**Table 2:** Onset potentials from cyclic voltammetry. Potentials vs. Ag/AgCl reference electrode.

Media	CO_2_	A/V	B/V	C/V	D/V
MEA	no	−0.5	−0.4	−0.48	−1.2
	yes	−0.21	−0.22	-	-
MDEA	no	−0.46	−0.27	−0.1	−0.97
	yes	−0.27	−0.27	-	-
MDEA/PZ	no	−0.44	−0.15	−0.47	−0.75
	yes	−0.17	−0.12	-	-
AMP	no	−0.52	−0.27	−0.51	−1.1
	yes	−0.25	−0.18	-	-
KCl	no	−0.4	−0.1	−0.06	−0.95

**Table 3:** Corrosion rates and time required to dissolve a 50 µm thick copper layer, calculated from corrosion current density data in Table 1.

		Dissolution into Cu(I)		Dissolution into Cu(II)	
Media	CO_2_	CR/mm y^−1^	Time/Days	CR/mm y^−1^	Time/Days
MEA	no	0.056	328	0.028	655
	yes	0.327	56	0.164	112
MDEA	no	0.007	2623	0.003	5242
	yes	0.023	787	0.012	1573
MDEA/PZ	no	0.009	1967	0.005	3931
	yes	0.021	874	0.010	1747
AMP	no	0.035	525	0.017	1048
	yes	0.179	102	0.089	204
KCl	no	0.056	328	0.028	655

## Data Availability

Data supporting the work are available in the Supplementary Materials.

## Supplementary Materials

The following supporting information can be downloaded at: https://www.mdpi.com/article/10.3390/ma17081825/s1, Figure S1: Schematic representing the electroplating setup, highlighting the positioning of the anode and cathode, maintained at a consistent distance of 95 mm; Figure S2: (**a**–**f**) Tafel extrapolation of potentiodynamic polarization of copper specimens in MEA. Scan rate 10 mV min^−1^; Figure S3: (**a**–**f**) Tafel extrapolation of potentiodynamic polarization of copper specimens in MDEA. Scan rate 10 mV min^−1^; Figure S4: (**a**–**f**) Tafel extrapolation of potentiodynamic polarization of copper specimens in MDEA-PZ. Scan rate 10 mV min^−1^; Figure S5: (**a**–**f**) Tafel extrapolation of potentiodynamic polarization of copper specimens in AMP. Scan rate 10 mV min^−1^; Figure S6: Linear polarization resistance of copper specimens in the different amine-based capture media. (**a**) MEA, (**b**) MDEA, (**c**) MDEA/PZ, (**d**) AMP. Scan rate 0.125 mV s^−1^; Table S1: Average cell resistances in the different amine solutions.
