# Supplementary material for: Electrochemical Characterization of Electrodeposited Copper in Amine CO2 Capture Media
Source: Materials (Basel). 2024 Apr 16;17(8):1825. doi: 10.3390/ma17081825 (PMC11051279; doi:10.3390/ma17081825)
Supplement: Supplementary file 1 [file materials-17-01825-s001.zip › materials-2912791-supplementary.pdf]

materials

Supplementary Information

Electrochemical Characterization of Electrodeposited Copper in Amine CO_2 Capture Media

Corentin Penot [1], Kranthi Kumar Maniam [1] and Shiladitya Paul [1,2,*]

[1] Materials Innovation Centre, School of Engineering, University of Leicester, Leicester LE1 7RH, UK; cp473@leicester.ac.uk (C.P.); km508@leicester.ac.uk (K.K.M.)
[2] Materials Performance and Integrity Technology Group, TWI, Cambridge CB21 6AL, UK
* Correspondence: shiladitya.paul@twi.co.uk

Citation: Penot, C.; Maniam, K.K.; Paul, S. Electrochemical Characterization of Electrodeposited Copper in Amine CO_2 Capture Media. *Materials* **2024**, *17*, 1825. https://doi.org/10.3390/ma17081825

Academic Editors: Georgi Kostadinov, Todor Penyashki and Mara Kandeva

Received: 26 February 2024
Revised: 5 April 2024
Accepted: 8 April 2024
Published: 16 April 2024

Copyright: © 2024 by the authors. Licensee MDPI, Basel, Switzerland. This article is an open access article distributed under the terms and conditions of the Creative Commons Attribution (CC BY) license (https://creativecommons.org/licenses/by/4.0/).

1. Electroplating of Copper

1.1. Materials

Copper sulfate pentahydrate (CuSO4.5H2O), sulfuric acid (H2SO4, 98% purity), and hydrochloric acid (HCl, 37% purity) were procured from Fischer Scientific (United Kingdom). Copper panels were acquired from Schloetter (United Kingdom).

1.2. Experimental Setup

1.2.1. Experimental Preparation

Initially, copper substrates were cut into 2.5 x 2.5 cm^{-2} sized specimens and polished using 600 and 1200 grade emery paper. Subsequently, they were rinsed in deionized water and activated in a 10 wt % HCl solution. Afterward, the samples were rinsed again with deionized water (DI) and utilized as the cathode for the copper deposition process. A copper plate measuring 5 x 10 cm^{-2} was employed as the anode.

1.2.2. Electroplating Solution

The electroplating solution was formulated by mixing 150 $g.L^{-1}$ of $CuSO_4.5H_2O$, 40 $mL.L^{-1}$ of H_2SO_4, and deionized water. Initially, DI water was warmed upto 45-50 deg C followed by the slow addition of 150 $g.L^{-1}$ of $CuSO_4.5H_2O$. Once the solution is well mixed, 40 $mL.L^{-1}$ of H_2SO_4 is added to the solution drop by drop until a homogeneous mixture is obtained. The resultant solution was cooled to laboratory temperature and employed as the electroplating solution

1.2.3. Electroplating Experiment

The electroplating experiment was conducted by applying a voltage of 0.63 V using a DC rectifier (10 A, 12 V, British Electricals Limited, United Kingdom) for a duration of 35 minutes. The distance between the anode and the cathode was maintained at 95 mm (as illustrated in Figure S1), and air agitation was employed to ensure the uniform distribution of concentrations near the cathode.

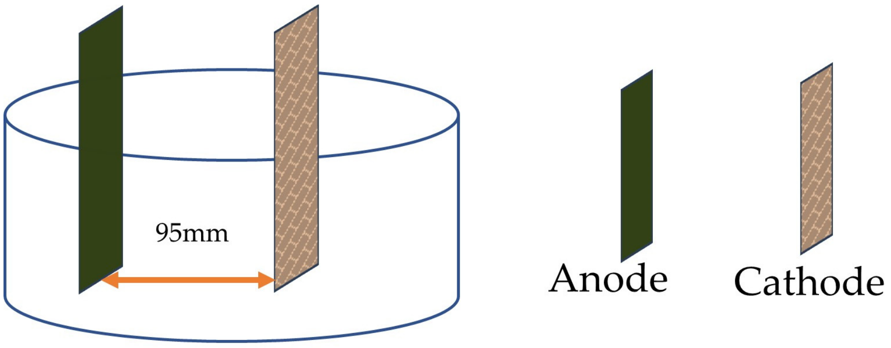

Figure S1. Schematic representing the electroplating setup, highlighting the positioning of the anode and cathode, maintained at a consistent distance of 95 mm.

2. Electrochemical Testing

To perform Tafel extrapolations, linear regression was performed on the anodic branch of the polarization plot (log(j) *vs*. potential). The corrosion current density (j_{corr}) was taken as the intersect between the linear regression and lowest current density recorded (at corrosion potential). All fits are presented bellow.

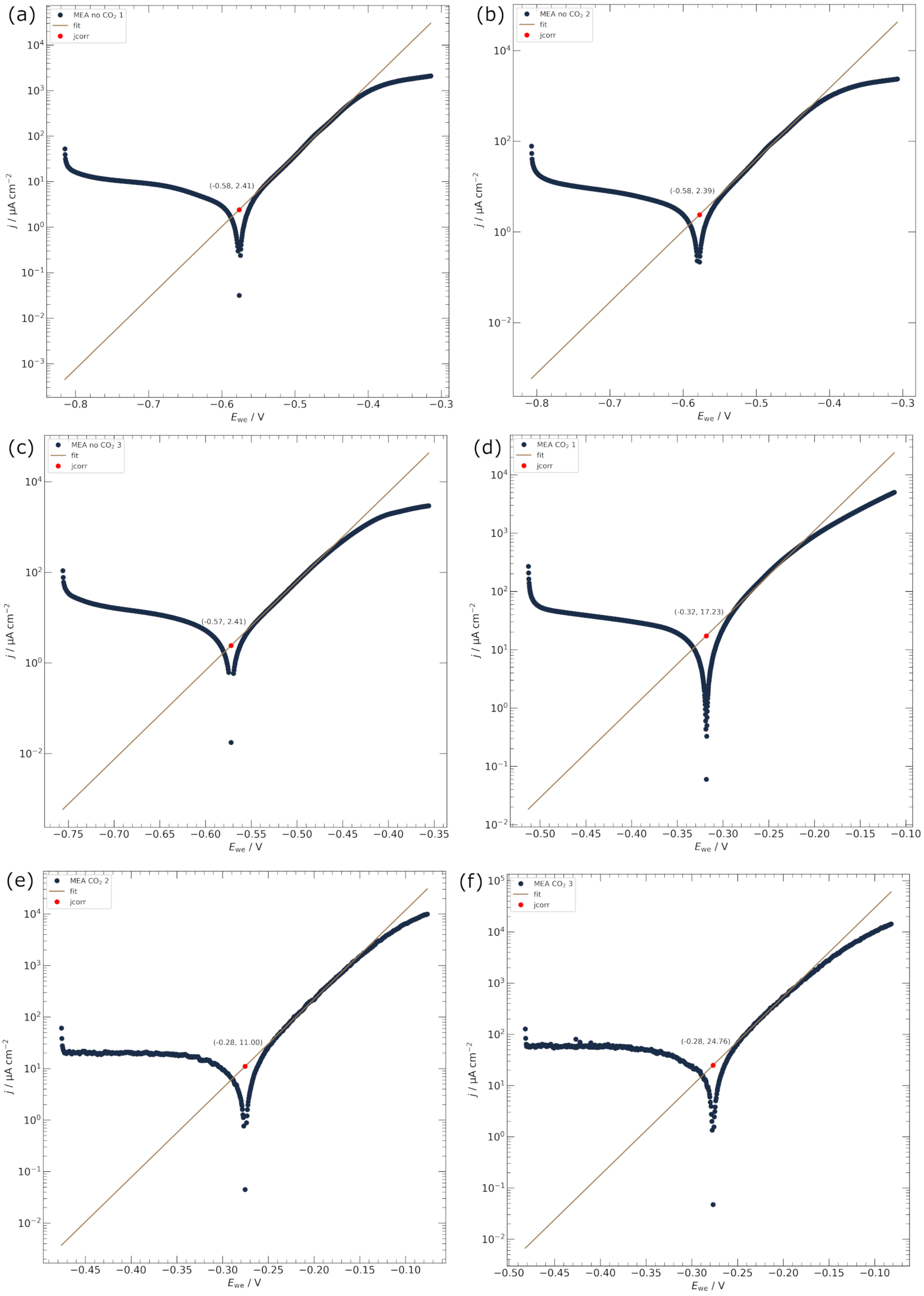

Figure S2. (**a**–**f**) Tafel extrapolation of potentiodynamic polarization of copper specimens in MEA. Scan rate 10 mV min-1.

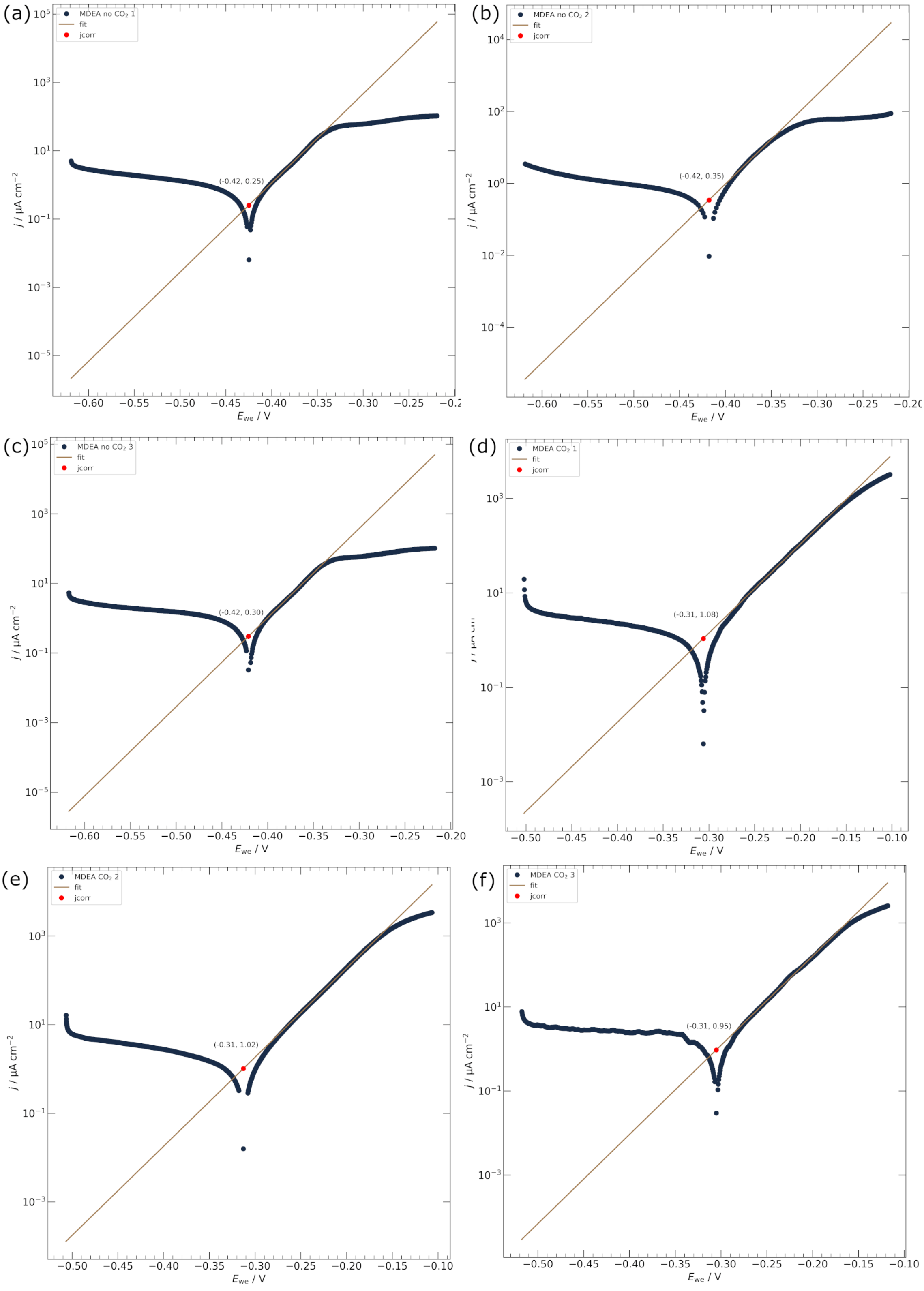

Figure S3. (**a**–**f**) Tafel extrapolation of potentiodynamic polarization of copper specimens in MDEA. Scan rate 10 mV min^{-1}.

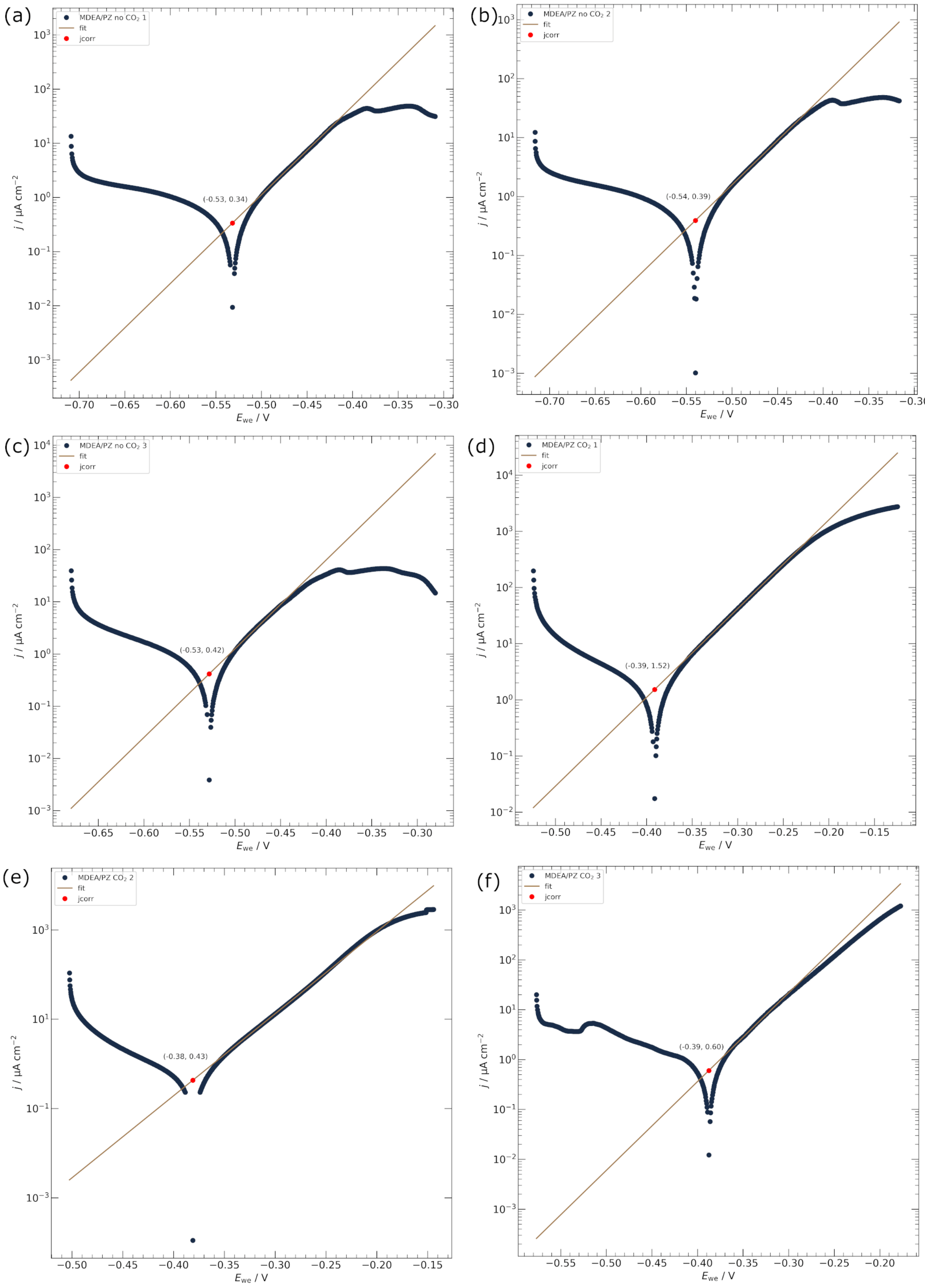

Figure S4. (**a**–**f**) Tafel extrapolation of potentiodynamic polarization of copper specimens in MDEA-PZ. Scan rate 10 mV min^{-1}.

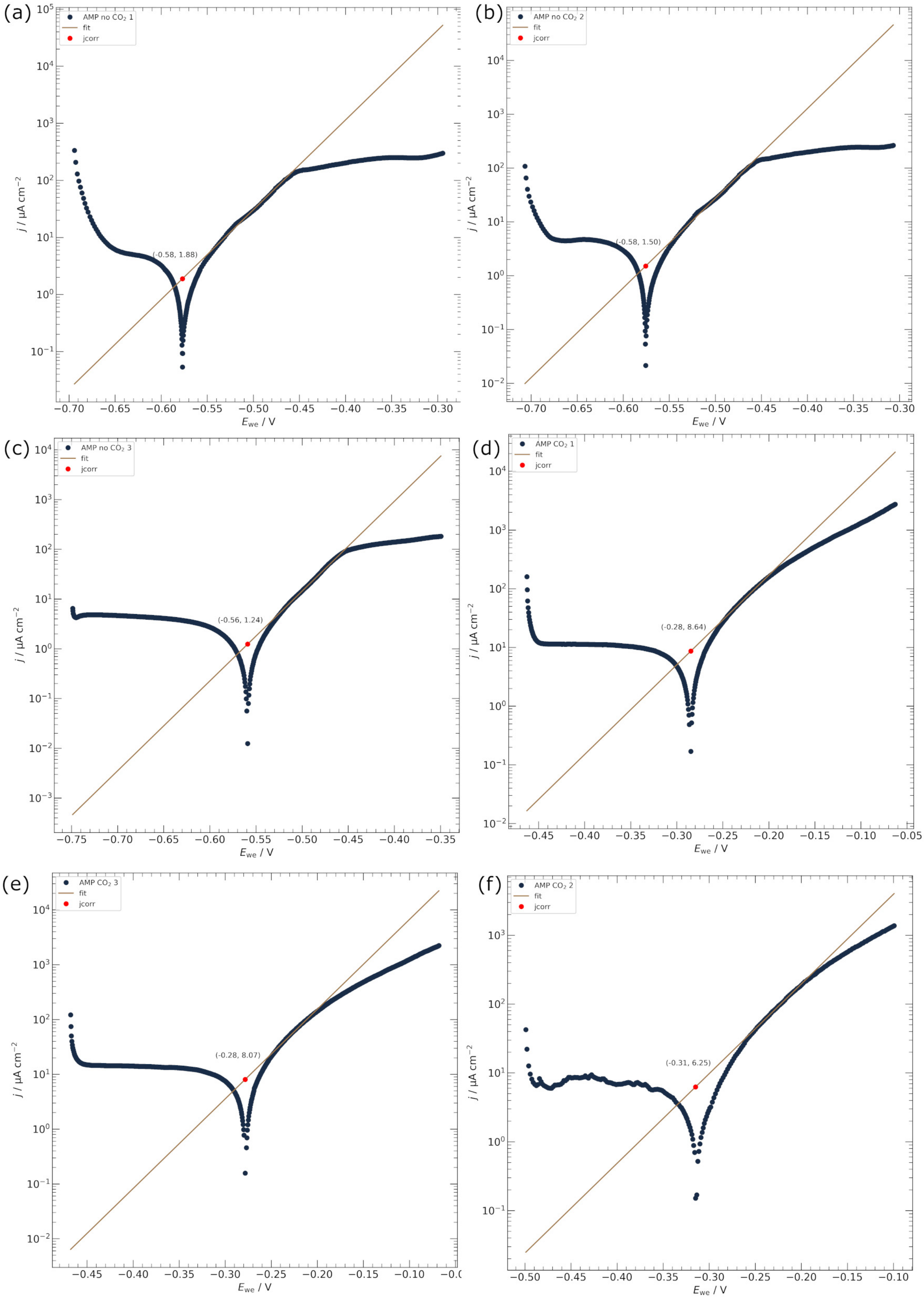

Figure S5. (**a**–**f**) Tafel extrapolation of potentiodynamic polarization of copper specimens in AMP. Scan rate 10 mV min^{-1}.

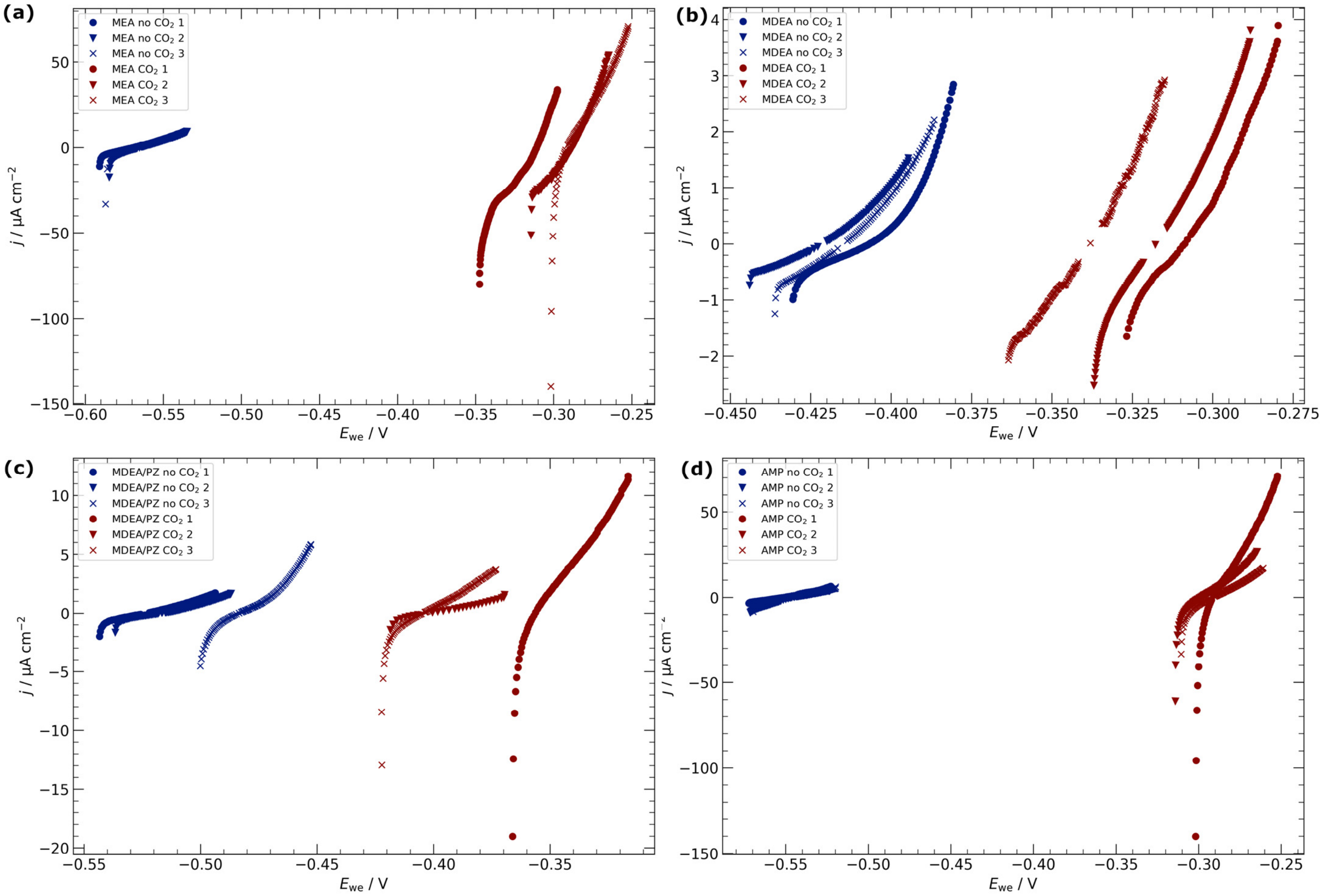

Figure S6. Linear polarization resistance of copper specimens in the different amine-based capture media. (**a**) MEA, (**b**) MDEA, (**c**) MDEA/PZ, (**d**) AMP. Scan rate 0.125 mV s^{-1}.

Table S1. Average cell resistances in the different amine solutions.

Media	CO_2	R_{cell} / Ω
MEA	no	7.8 ± 0.6
	yes	2.0 ± 0.2
MDEA	no	17.4 ± 2.7
	yes	8.3 ± 0.7
MDEA/PZ	no	18.7 ± 9.0
	yes	12.6 ± 2.4
AMP	no	7.5 ± 1.2
	yes	4.4 ± 0.2
KCl	no	2.4 ± 2.1
